# The ClassA Framework: HRV Based Assessment of SNS and PNS Dynamics Without LF-HF Controversies

**DOI:** 10.3389/fphys.2019.00505

**Published:** 2019-04-30

**Authors:** Tricia Adjei, Wilhelm von Rosenberg, Takashi Nakamura, Theerasak Chanwimalueang, Danilo P. Mandic

**Affiliations:** ^1^Communications and Signal Processing, Department of Electrical and Electronic Engineering, Imperial College London, London, United Kingdom; ^2^Department of Biomedical Engineering, Faculty of Engineering, Srinakharinwirot University, Nakhon Nayok, Thailand

**Keywords:** autonomic nervous system, heart rate variability, LF, HF, second-order-difference-plot

## Abstract

The powers of the low frequency (LF) and high frequency (HF) components of heart rate variability (HRV) have become the de facto standard metrics in the assessment of the stress response, and the related activities of the sympathetic nervous system (SNS) and the parasympathetic nervous system (PNS). However, the widely adopted physiological interpretations of the LF and HF components in SNS /PNS balance are now questioned, which puts under serious scrutiny stress assessments which employ the LF and HF components. To avoid these controversies, we here introduce the novel Classification Angle (ClassA) framework, which yields a family of metrics which quantify cardiac dynamics in three-dimensions. This is achieved using a finite-difference plot of HRV, which displays successive rates of change of HRV, and is demonstrated to provide sufficient degrees of freedom to determine cardiac deceleration and/or acceleration. The robustness and accuracy of the novel ClassA framework is verified using HRV signals from ten males, recorded during standardized stress tests, consisting of rest, mental arithmetic, meditation, exercise and further meditation. Comparative statistical testing demonstrates that unlike the existing LF-HF metrics, the ClassA metrics are capable of distinguishing both the physical and mental stress epochs from the epochs of no stress, with statistical significance (Bonferroni corrected *p*-value ≤ 0.025); HF was able to distinguish physical stress from no stress, but was not able to identify mental stress. The ClassA results also indicated that at moderate levels of stress, the extent of parasympathetic withdrawal was greater than the extent of sympathetic activation. Finally, the analyses and the experimental results provide conclusive evidence that the proposed nonlinear approach to quantify cardiac activity from HRV resolves three critical obstacles to current HRV stress assessments: (i) it is not based on controversial assumptions of balance between the LF and HF powers; (ii) its temporal resolution when estimating parasympathetic dominance is as little as 10 s of HRV data, while only 60 s to estimate sympathetic dominance; (iii) unlike LF and HF analyses, the ClassA framework does not require the prohibitive assumption of signal stationarity. The ClassA framework is unique in offering HRV based stress analysis in three-dimensions.

## 1. Introduction

The endeavor to objectively discern states of stress from rest using only heart rate variability (HRV) has long been a subject of research. Despite the existence of many *qualitative* stress assessments, such as questionnaires, the inherent subjectivity of these measures restricts their use in practical applications. For example, a subjective measure of stress cannot be relied upon to assess whether a soldier is fit for deployment, neither can a subjective measure be used to assess if subjects with poor verbalization (such as neonates) are experiencing excessive levels of stress or trauma. The ability to objectively and quantitatively identify an individual's stress state would therefore facilitate the creation of readily deployable tools for stress monitoring and management in every-day life.

Attempts to *quantitatively* identify stress from physiological signals have included temporal (Castrillión et al., 2017), spectral (Montano et al., 1994), and nonlinear (Vuksanovic and Gal, 2007) measures, however, conventional algorithms have not been able to achieve an accurate and precise discernment of stress states from rest. This is a reflection of the complexity of the human stress response, for which the causes are manifold, and the manifestations are yet to be fully understood. A pioneer in stress research, Cannon (1915), was the first to coin the phrase “fight-or-flight” to describe a generic physiological response to combat stress. Fight-or-flight encapsulates the phenomenon of physiological energization to overcome a stressor, and is largely driven by the sympathetic nervous system (SNS). However, it is now well established that the responses to stress are not exclusively sympathetic; stress reactions driven by the parasympathetic nervous system (PNS) include the production of tears and the emptying of the bladder (Berntson et al., 1991; Avnon et al., 2004). Yet, despite the known PNS responses to stressors, many of the traditional quantitative measures of stress are dependent upon detecting changes in the activity of the SNS; for example, heart rate, the most basic measure of stress, is expected to rise as a result of sympathetic activation. Other traditional measures of stress include temporal methods, such as the standard deviation of beat-to-beat intervals (SDNN), the spectral components of HRV, and nonlinear signal measures (Malik et al., 1996).

The spectral analyses of HRV to determine SNS and PNS dynamics are the most widely used technique in the quantitative assessment of stress, and have been inspired by the known differences in the speed of PNS and SNS activations. Given that the parasympathetic nerves elicit a much faster ( ≤ 1 s) response than those of sympathetic nerves (≥ 5 s) (Nunan et al., 2010), the power of the 0.04–0.15 Hz and the 0.15–0.4 Hz bands of HRV, respectively, defined as the powers of the low frequency (LF) and high frequency (HF) bands, have been used to characterize the stress response. The use of the LF and HF powers in stress analysis dates back to work published by Montano et al. (1994), where the orthostatic tilt of study participants was found to positively correlate with the power in the LF band, and negatively correlate with the power in the HF band. It was thus concluded that the power of the LF band reflected activity of the sympathetic nervous system (PNS), whilst the power of the HF band reflected the activity of the parasympathetic nervous system (PNS) (Montano et al., 1994). These findings were supported in many subsequent studies which used nerve activities as references of SNS and PNS activities (Pagani et al., 1997). However, in spite of growing concerns over the validity of the use of spectral powers to assess autonomic dynamics (Eckberg, 1997), the LF and HF components were rapidly accepted to become the de facto measures of stress. The widespread use of the LF and HF powers continued until Billman (2013) produced a thorough critique of the method, highlighting that the SNS was not the sole contributor to the LF peak of HRV spectra, and that it is the PNS which is the biggest contributor to the variability of the LF peak. Billman (2013) also reported that the PNS is not the sole contributor to the HF peak in HRV spectra. Other findings have suggested that other physiological modalities, such as slow breathing, influence the LF component of HRV (Brown et al., 1993). Typical breathing rates have been found to influence the HF component of HRV, whilst the myogenic contractions of the muscles in blood vessels have also been found to influence the LF band in HRV (Kenwright et al., 2008). These findings provide conclusive evidence that the use of the powers of the LF and HF components to assess SNS and PNS activities is in general erroneous, and not advisable for future eHealth applications.

Nonlinear measures are now increasingly employed to identify stress. The rise in the popularity of nonlinear measures can be attributed to research findings that physiological signals can contain nonlinearities (Gautama et al., 2004).

Measures of entropy are common nonlinear methods of signal analysis, and the measurement of signal entropy is analogous to the assessment of irregularity in a signal; Bornas et al. (2006), Vuksanovic and Gal (2007), Williamon et al. (2013), and Chanwimalueang et al. (2016) all reported stress-induced reductions in signal entropy. Although such measures may be able to identify instances of stress, they can be computationally inefficient, or leave poor temporal resolution, in addition to not indicating the physiological mechanisms which lead to the stress response. Symbolic measures of stress have also been proposed in which the occurrence of specific symbols, or patterns, in a signal are counted; Porta et al. (2007) introduced a symbolic measure which successfully identified SNS and PNS dynamics from HRV during tilt tests. However, Porta et al. (2007) also pointed out that despite their reporting of SNS dynamics, the length of symbols employed in their analysis may have been too short to accurately capture long-range SNS dynamics.

Model-based algorithms have also been recently introduced to assess autonomic responses to tilt; Valenza et al. (2018) introduced two measures to discern SNS and PNS dynamics, these were derived using kernels generated from a dataset of HRV signals. However, the accuracy of such model-based approaches is dependent upon the fit of the model (Valenza et al., 2018).

In light of the above described inaccuracies associated with current stress measures, we set out to investigate the usefulness of second-order-difference-plots to assess cardiac deceleration and acceleration from HRV signals. The developed framework aims to assess the spread of the finite-differences in HRV series, and introduces a family of stress metrics, collectively termed Classification Angle (ClassA). A multiscale analysis is employed to account for the long-range variations in SNS activity and the short-range variations of the PNS; this means that the metrics pertaining to the SNS and PNS do not assume balance, and are therefore able to provide insight into the separate dynamics of the SNS and PNS. The Classification Angle framework is comprised of four metrics, three of which offer a three-dimensional assessment of stress, capable of separating different stress states:

- **Real Angle Sum (RAS)**. The mean of the sum of the angles between HRV rates of change and the abscissa in a scatter plot of finite-differences;
- **Cardiac Deceleration Proportion (**${\text{P}}^{{}_{Q1}}$**)**. The proportion of HRV rates of change which fall into the first quadrant of a scatter plot of finite-differences;
- **HRV Balance Proportion (**${\text{P}}^{{}_{Q2,4}}$**)**. The proportion of HRV rates of change which fall into the second and fourth quadrants of a scatter plot of finite-differences;
- **Cardiac Acceleration Proportion (**${\text{P}}^{{}_{Q3}}$**)**. The proportion of rates of change of coarse-grained HRV which fall into the third quadrant of a scatter plot of finite-differences.

For rigor, we perform a comprehensive comparison of the results from the ClassA analyses of HRV signals, acquired from ten males during a standardized stress test, to those from traditional temporal, spectral and nonlinear analyses. This serves to conclusively demonstrate the superior statistical performance of ClassA in the ability to discern stress epochs from rest epochs; two of the ClassA metrics are shown to be significantly more accurate in the characterization of stress than the current state-of-the-art measures of heart rate, the standard deviation of beat-to-beat intervals, the power of the LF component of HRV, the power of the HF component of HRV, and the nonlinear measures of sample entropy and permutation entropy.

## 2. Classification Angle

### 2.1. Second-Order-Difference-Plots

The Classification Angle framework is based on a scatter plot of HRV finite-differences, a second-order-difference-plot (SODP), first introduced by Cohen et al. (1996). The original SODP is a plot of (*x*(*n* + 2) − *x*(*n* + 1)) against (*x*(*n* + 1) − *x*(*n*)), where *x*(*n*) is a point in an HRV series at a time instant *n*. The SODP is therefore a plot of successive HRV rates of change against one another, and its physical interpretation is the correlation between successive HRV rates (Cohen et al., 1996; Kamath, 2012; dos Santos et al., 2015). The SODP was first introduced as a means to assess the degree of variability within HRV signals, and has been found to be more robust than the more commonly used Poincaré plot (Cohen et al., 1996). Extensions of the SODP, such as the measure of dispersion called the central tendency measure have also been found to be able to characterize other physiological signals, such as intracranial pressure signals (Cohen et al., 1996; Sanatmarta et al., 2012). Furthermore, as it has been found that HRV can be nonlinear (Gautama et al., 2004), the use of standard “Gaussian" mathematical models is not guaranteed to provide the best descriptors of HRV dynamics (Cohen et al., 1996), and HRV should therefore be analyzed using a nonlinear method, such as an SODP (Cohen et al., 1996; dos Santos et al., 2015; Makowiec et al., 2017). The spectral methods typically used in HRV analyses (to compute the LF and HF powers) are however linear.

**Figure 1:**
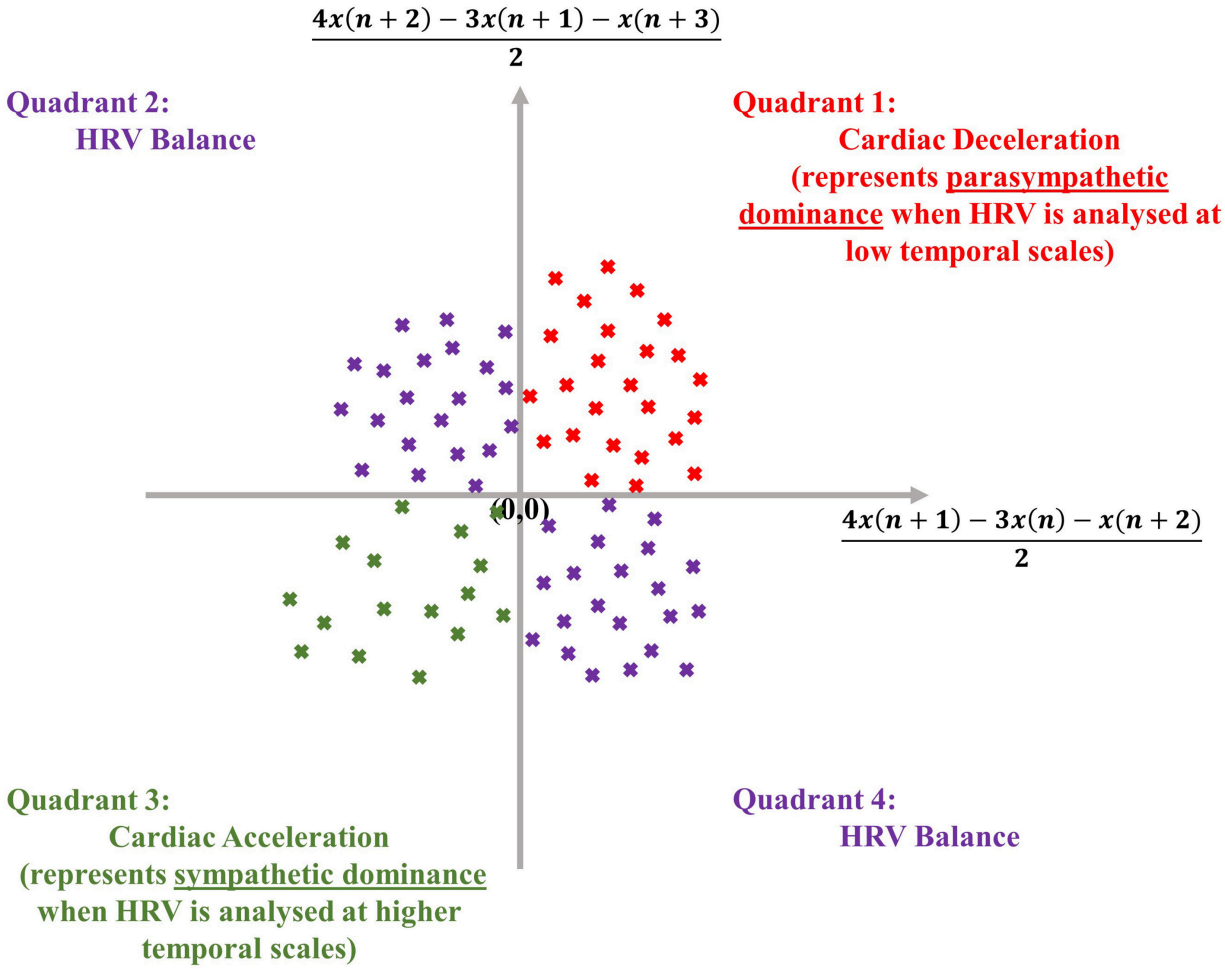
Physiological interpretation of the quadrants of the second-order-difference-plot (inspired by Kamath, 2012), employed in the Classification Angle (ClassA) framework.

The dispersion of points in an SODP, as shown later, also conveys physical meaning, as the quadrants of an SODP can be interpreted physiologically; the first quadrant represents cardiac deceleration, the second and fourth quadrants represent a balanced HRV sequence, whilst the third quadrant represents cardiac acceleration (Kamath, 2012) (see Figure 1). The physiological interpretation of the SODP quadrants, at two temporal scales, forms the basis of the ClassA framework.

The use of a data-point dispersion method (although not an SODP) in the assessment of autonomic functioning is supported by findings from Makowiec et al. (2017). Makowiec et al. (2017) used a visualization network tool, similar to a Poincaré plot, to assess HRV increments and autonomic drive, and reported that autonomic supervision caused larger increments in HRV, when compared to hearts functioning without autonomic supervision.

### 2.2. Classification Angle Framework

Finite differencing is a means to estimate the first derivative of a signal (Mandic and Goh, 2009), with the first-difference method employed by Cohen et al. (1996), (*x*(*n* + 1) − *x*(*n*)), being the most common. However, the use of (*x*(*n* + 1) − *x*(*n*)) is not as accurate as other approximations of the derivative; for example, an SODP using the three-point forward approximation of [(4*x*(*n* + 2) − 3*x*(*n* + 1) − *x*(*n* + 3))/2] against [(4*x*(*n* + 1) − 3*x*(*n*) − *x*(*n* + 2))/2] is a more accurate approximation (Butt, 2010). Therefore, we employ the three-point forward derivative approximation in the ClassA framework, which is given in Framework 1. To summarize the framework, ClassA computes the proportion of points in the quadrants of an SODP for an HRV series at two temporal scales.

Signal fluctuations over different temporal scales are common for physiological systems (Zhang, 1991; Costa et al., 2002), and signal analysis at two different temporal scales is of particular relevance in HRV analysis due to the faster speed of parasympathetic nerves compared to sympathetic nerves (Nunan et al., 2010). The coarse-graining procedure employed in the ClassA framework consists of averaging adjacent data-points in nonoverlapping windows, where a signal, *x*(*n*) of length *N*, is accessed at temporal scale, τ, to produce a coarse-grained signal, *y*(*i*), with the indices of the coarse-grained signal denoted by *i*, as shown below (Costa et al., 2002).

(1) $$\begin{array}{r} y\left(i\right)=\frac{1}{\tau }\overset{i\tau }{\underset{n=\left(i-1\right)\tau +1}{\sum }}x\left(n\right)\;1\leq i\leq N/\tau \end{array}$$

The signal at its original temporal scale of one retains its original length; it should be treated as beat-to-beat HRV and contains short-range PNS dynamics. The longer-range SNS dynamics are evident at higher temporal scales of the HRV signal, however, as the process of coarse-graining inevitably shortens a signal, a longer window of analysis must be used to analyse the signal at the higher temporal scale. Therefore, as a rule, the HRV signal must be coarse-grained to a scale that is sufficiently high to capture longer range SNS dynamics, but which also retains a comparable number of data-points to that of the windowed signal at its original temporal scale. The points in the first quadrant, and the points in the second and fourth quadrants of the SODP for an HRV signal are found at the original temporal scale to represent the degree of PNS dominance and the degree of HRV balance, respectively. Whilst the points in the third quadrant of the SODP for the HRV signal at a higher temporal scale represent the degree of SNS dominance, and thus, is at a different temporal resolution to the measure of PNS dominance.

**Figure 2:**
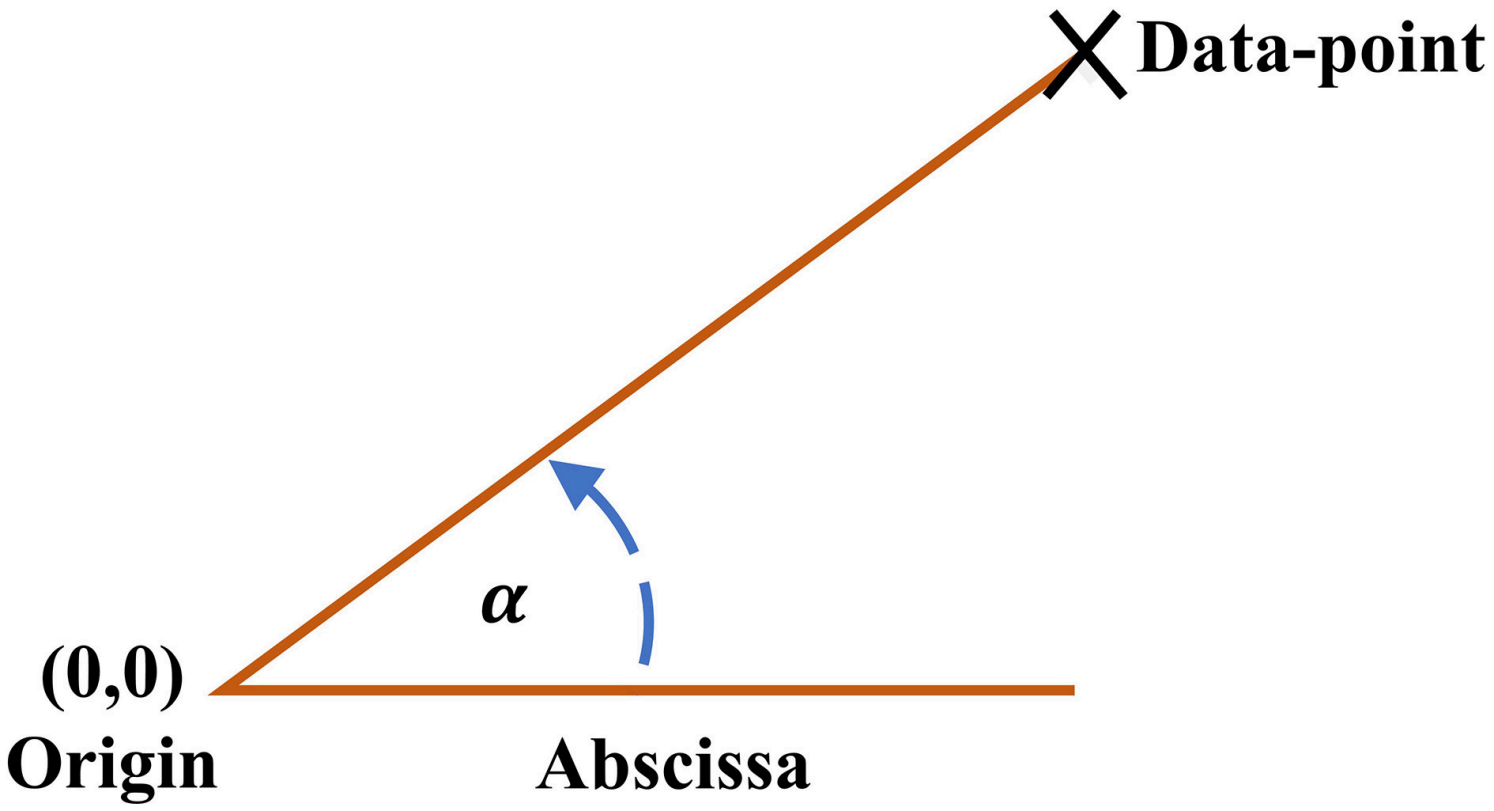
Angle between each data-point on the Classification Angle (ClassA) second-order-difference-plot and the abscissa.

The ClassA framework also allows for the computation of a trend detection measure, the Real Angle Sum (RAS), which is the mean of the sum of the angles in the anti-clockwise direction between every data-point in the SODP and the abscissa; for illustration of the principle see Figure 2. The Real Angle Sum is measured in degrees (°) and is interpreted using the quadrants of an SODP; the value of RAS indicates the overall trend in data where a RAS between 0 and 90° indicates a predominantly increasing sequence, a RAS between 90–180° and 270–360° designates a predominantly balanced sequence, and a RAS between 180 and 270° reflects a predominantly decreasing sequence. We shall use RAS to quantify stress levels with a single value, where we hypothesize that RAS will tend to a value in the range of 180–270° during stress.

**Frame Work 1:** Classification Angle (ClassA) Framework.

Create a scatter plot of first differences within HRV by plotting [(4*x*(*n*+2)−3*x*(*n*+1)−*x*(*n*+3))/2] against [(4*x*(*n*+1)−3*x*(*n*)−*x*(*n*+2))/2] for a signal, *x*. Compute, in the anti-clockwise direction, the angle, α_*n*_, that each point in the scatter plot makes with the abscissa, such that data-points in the first quadrant of the scatter plot will make acute angles with the abscissa, those in the second quadrant will make obtuse angles, and those in the third and fourth quadrants will make reflex angles. Sum the angles, α_*n*_, computed in Step 2, and divide the total by the number of data-points, *N*, in the HRV signal. Designate this total as the Real Angle Sum (RAS), $RAS=\frac{\sum \alpha_{n}}{N}$. Count the number of points in the scatter plot which fall within the first quadrant, and denote this by $Q^{{}_{1}}N$. Divide $Q^{{}_{1}}N$ by *N* to find the proportion of the total number of points that lie in the first quadrant, $P^{{}_{Q1}}$, that is $P^{{}_{Q1}}=\frac{Q^{{}_{1}}N}{N}$. Repeat Steps 4 and 5 to compute the proportion of points in the second and fourth quadrants together, $P^{{}_{Q2,4}}$. Course-grain the HRV signal to access the signal at a higher temporal scale, and repeat Steps 4 and 5 to compute the proportion of points in the third quadrant, $P^{{}_{Q3}}$. The metrics RAS, $P^{{}_{Q1}}$, $P^{{}_{Q2,4}}$ and $P^{{}_{Q3}}$ are the four outputs of ClassA. Create a three-dimensional plot of $P^{{}_{Q2,4}}$ against $P^{{}_{Q3}}$ against $P^{{}_{Q1}}$ to identify different states of stress. The overall trend in the data is indicated by RAS.

## 3. Materials and Methods

### 3.1. Subjects

The performance of the ClassA framework was verified on 10 HRV signals, recorded from 10 males, with a mean age of 28.6 (standard deviation: 5.6, range: 23–38). A dataset size of 10 is supported by Ristic-Djurovic et al. (2018), who report that the minimum dataset size required to produce statistically significant results in a biomedical study is nine.

### 3.2. Stress Test

**Figure 3:**
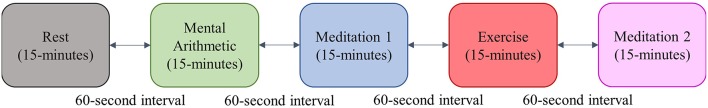
Protocol for the stress test employed in this study.

The participants in this study completed a standardized stress test in pairs. The test was inspired by the Trier Social Stress Test (Birkett, 2011), and consisted of 15 min of rest, 15 min of a mental arithmetic test, 15 min of meditation, 15 min of a step-exercise, and a final 15 min of meditation. To increase the level of stress induced by the arithmetic test, the subjects were told that the test was a competition, and that their scores would be recorded. There was a 1-min interval between each test epoch; an outline of the test protocol is shown in Figure 3.

The stress test was explained both verbally and in writing, and full consent was obtained from all subjects. Approval for physiological sensing for stress assessment has been granted by the Imperial College Healthcare Trust Clinical Governance Department.

### 3.3. Data Acquisition

The electrocardiograms (ECGs) of the subjects were recorded whilst they completed the stress test, using a custom-made portable data-logger, called the iAmp (Goverdovsky et al., 2017; Kanna et al., 2018). The ECGs were recorded using adhesive surface electrodes in the Lead I configuration, at a sampling rate of 1000 Hz.

All signal analyses were undertaken using the MATLAB programming environment. The R-peaks within the ECGs were detected to extract HRV, using the robust algorithm introduced in Chanwimalueang et al. (2015); the HRV signals were then re-sampled at 4 Hz. It has been suggested that the re-sampling of HRV can alter the frequency content of HRV power spectra, however this effect is minimized when signals are clean (Moody, 1993).

The ClassA framework was applied to each recorded signal, and the results were segmented in accordance with the test epochs. For the purposes of comparison, temporal, spectral and nonlinear analyses were also undertaken, to compute heart rate, the SDNN, the powers of the LF and HF components, sample entropy and permutation entropy from the HRV signals.

The ClassA analyses to obtain $P^{{}_{Q1}}$, $P^{{}_{Q2,4}}$ and RAS were undertaken within a 10-s window, with a 1-s increment, whilst the ClassA analyses to obtain $P^{{}_{Q3}}$ were computed in a 60-s window with a 1-s increment, whereby HRV signals were coarse-grained to a τ of seven. Therefore, regarding the respective windows of analyses, $P^{{}_{Q1}}$, $P^{{}_{Q2,4}}$ and RAS were computed from 40 data-points, and $P^{{}_{Q3}}$ was computed from 34 data-points. All other analyses were undertaken within a 5-min window, with a 1-s increment. The use of a 5-min window is in accordance with the recommendations made by the Task Force of the European Society of Cardiology and the North American Society of Pacing and Electrophysiology, for the accurate computation of the traditional HRV metrics (Malik et al., 1996). Within each window of analysis, outliers in the HRV were replaced with the median of the interquartile range of the data; outliers were defined as data values which are either 1.5 times smaller than the 25th percentile of the data, or 1.5 times greater than 75th percentile of the data.

### 3.4. Temporal Analyses

Heart rate (HR) in beats-per-minute (*bpm*) was computed from the HRV signals using Equation (2), where *x*(*n*) and *N*, respectively, designate an HRV data-point and the total number of data-points in the window of analysis, that is

(2) $$\begin{array}{c} HR=\frac{\sum_{n=1}^{N}\left(\frac{60}{x(n)}\right)}{N} \end{array}$$

The SDNN in milliseconds (*ms*) is expected to decrease in the presence of stress (Kim et al., 2018), and was computed as

(3) $$\begin{array}{c} SDNN=\sqrt{\frac{\sum_{n=1}^{N}{\left(x(n)-\bar{x(n)}\right)}^{2}}{N-1}} \end{array}$$

where $\bar{x\left(n\right)}$ represents the mean.

### 3.5. Spectral Analyses

The spectral measures of the absolute powers of the low frequency (0.04–0.15 Hz) and high frequency (0.15–0.4 Hz) components of HRV were computed, and are denoted by aLF and aHF, respectively, with units of milliseconds squared (*ms*^2^) (Burr, 2007). The powers were estimated using the MATLAB function “bandpower,” which uses the periodogram method.

The periodogram is a method to estimate spectral power from the Discrete Fourier Transform (DFT) of a signal (the DFT represents a discrete signal as a periodic signal) (Akin and Kiymik, 2000). The Fourier coefficients from the DFT are then used to determine the frequency components of the signal and the respective powers (Akin and Kiymik, 2000).

A Hamming window of the same length as the signal was used to compute the spectral powers; Equations 4 and 5 describe the computation of the periodogram (Akin and Kiymik, 2000), where the DFT, *X*(ω_*k*_), for a signal, *x*, is computed for time, *t*, where ω_*k*_ denotes the angular frequency, *j* denotes the imaginary number and *N* represents the length of the signal.

(4) $$\begin{array}{r} X\left(\omega_{k}\right)=\overset{N-1}{\underset{t=0}{\sum }}x\left(t\right)e^{\frac{-j\omega_{k}t}{N}} \end{array}$$

The spectral power, *P*(*f*_*k*_), is computed for a frequency, *f*_*k*_, with *f*_*s*_ as the signal sampling frequency (Akin and Kiymik, 2000) as follows

(5) $$\begin{array}{r} P\left(f_{k}\right)=\frac{f_{s}}{N}|X\left(k\right){|}^{2} \end{array}$$

However, it must be noted that absolute values of the LF and HF power are greatly affected by the total power in an HRV signal (Malik et al., 1996). For example, although tachycardia is caused by sympathetic excitation, it can also cause a decrease in total power, and hence also reduces the absolute LF power (Malik et al., 1996); aLF and aHF powers are therefore often normalized.

The absolute powers of LF and HF were normalized by the power of the 0.04–0.5 Hz band, *N*_*p*_, as shown in Equations (6) and (7). The normalized low frequency and high frequency powers will be referred to as nLF and nHF, and are reported as percentages.

(6) $$\begin{array}{r} nLF=\frac{aLF}{N_{p}} \end{array}$$

(7) $$\begin{array}{r} nHF=\frac{aHF}{N_{p}} \end{array}$$

The power in the 0.04–0.5 Hz band of HRV, *N*_*p*_, was used for the normalization for the following reasons: (i) there is no clear physiological interpretation for frequencies below 0.04 Hz (Malik et al., 1996) and (ii) the minimal rate at which the heart muscle can contract is typically 1 Hz (60 beats per minute), and thus, in accordance with the Nyquist theorem, the useful information content of HRV is contained below 0.5 Hz (Kuusela, 2004).

### 3.6. Nonlinear Analyses

The results from ClassA were also compared to the results from two nonlinear measures; sample entropy and permutation entropy. Vuksanovic and Gal (2007), Williamon et al. (2013), and Chanwimalueang et al. (2016) have all reported stress induced decreases in the entropy of HRV.

#### 3.6.1. Sample Entropy

Sample entropy (SE) was introduced by Richman and Moorman (2000) as an improvement to approximate entropy from Pincus (1991). Sample entropy can be described as the negative natural logarithm of the likelihood that a sequence of length *m* from a signal, *x*(*n*), will remain similar, within a given tolerance, *r*, to the sequence of length (*m* + 1) (Richman and Moorman, 2000). Sample entropy is limited by its dependence on the user-defined parameters of *m* and *r*; SE is often unstable when *m* is larger than four, and the tolerance *r* is best recommended to be a multiple of the standard deviation (Pincus and Keefe, 1992), meaning it is not suited to nonstationary physiological signals. The SE computation employed in this study is outlined in Algorithm 1 in the Appendix. An embedding dimension of 2 and a tolerance of 0.15 multiplied by the standard deviation were used.

#### 3.6.2. Permutation Entropy

Permutation entropy (PE) is a symbolic measure similar to that employed by Porta et al. (2007), which measures the occurrence of unique patterns, of length *m*, in a signal. The algorithm was introduced by Bandt and Pompe (2002) as a robust measure of regularity. The embedding dimension *m* is the only user defined parameter in the PE algorithm, and is recommended to be a value in the range of 3 to 7, such that *m*! ≤ *N* (Bandt and Pompe, 2002). The computation of PE is described in Algorithm 2 in the Appendix (Bandt and Pompe, 2002; Bian et al., 2012). An embedding dimension of 6 was used.

As described in section 3.2 and shown in Figure 3, the standardized stress test used in this study consisted of the five epochs of rest, mental arithmetic, meditation 1, exercise and meditation 2, which means that the epoch of mental stress (arithmetic) is preceded by an epoch of no stress, and the epoch of physical stress (exercise) is also preceded by an epoch of no stress. The results from the above described metrics were then segmented to correspond with the test epochs.

The mean of the ClassA metrics of RAS, $P^{{}_{Q1}}$, $P^{{}_{Q2,4}}$, and $P^{{}_{Q3}}$, and the mean HR, SDNN, aLF, aHF, nLf, nHF, SE and PE from all five epochs of recorded data were compared using the multiple comparisons Kruskal-Wallis test, which indicates whether the computed measures from the five test epochs came from distributions with the same mean (Kanji, 2006). The mean metrics from the arithmetic epochs were then compared to those from the rest epochs, and the mean metrics from the exercise epochs were compared to those from the meditation 1 epochs in a pairwise comparison, to ensure that the results from each stress epoch were compared to those from an epoch of no stress. The Kruskal-Wallis test, with a significance level of 0.05 was used to assess these comparisons, and a Bonferroni-corrected statistical significance level of 0.025 was assumed in the pairwise Kruskal-Wallis tests; a corrected significance level of 0.05/2, accounts for two pairwise comparisons (rest vs. arithmetic, and meditation 1 vs. exercise).

Correlations between the ClassA metrics and the traditional measures were computed using Spearman's rho; a Bonferroni-corrected statistical significance level of 0.0016 is assumed to account for 32 pairwise comparisons between the four ClassA metrics and the eight traditional measures.

## 4. Results

### 4.1. Multiple Comparisons of All Five Epochs Within the Stress Test

The *p*-values from the Kruskal-Wallis multiple comparison tests of the traditional HRV measures are shown in Table 1. Multiple comparisons of the HR results produced a statistically significant *p*-value of 0.013, whilst the corresponding *p*-value for SDNN was only marginally significant at 0.057. The significant *p*-values are shown in bold. Median HR increased in response to stress, peaking during exercise, at 92 beats per minute (*bpm*), and was joint lowest during rest and meditation 1, at 75 *bpm*. Median SDNN peaked during the arithmetic test and was at its lowest during exercise, at 74 *ms* and 40 *ms*, respectively. Median aLF reached a peak at 0.0011 *ms*^2^ during the arithmetic test, and was lowest during exercise, at 0.00044 *ms*^2^, whilst Median aHF peaked during rest, at 0.00036 *ms*^2^, and was lowest at 0.00012 *ms*^2^, during exercise. Conversely, median nLF peaked during arithmetic at 73%, and was lowest at 61%, during rest. Median nHF peaked during rest at 37%, and was at its lowest at 23% during exercise. Median SE peaked at 0.70 during exercise and was at a minimum during arithmetic at 0.59. Median PE peaked at 0.57 during exercise, and was at a minimum during meditation 1, at 0.46.

**Table 1:** The *p*-values from the Kruskal-Wallis tests of the comparisons of the traditional HRV measures across all five test epochs.

**Traditional HRV Measures: *p*-values**	
**Measure**	***p*-value**
HR (*bpm*)	**0.013**
SDNN (*ms*)	0.057
aLF (*ms*^2^)	0.41
aHF (*ms*^2^)	0.13
nLF (%)	0.37
nHF (%)	0.09
SE	**0.026**
PE	**9.3 × 10^−5^**

*Significant p-values are shown in bold (p ≤ 0.05)*.

The *p*-values from the Kruskal-Wallis multiple comparison tests of the ClassA metrics are shown in Table 2. The multiple comparisons of the $P^{{}_{Q1}}$, $P^{{}_{Q2,4}}$, $P^{{}_{Q3}}$ and RAS results all produced statistically significant *p*-values of 7.4 × 10^−5^, 1.2 × 10^−5^, 0.002, and 1 × 10^−4^, respectively. These values are shown in bold in Table 2. Median $P^{{}_{Q1}}$ peaked at 0.44 during rest, and was lowest at 0.36 during exercise. Median $P^{{}_{Q2,4}}$ peaked during exercise, at 0.26, and was joint lowest at 0.18 during the rest and meditation 1 epochs. Median $P^{{}_{Q3}}$ peaked at 0.27 during exercise, and was lowest at 0.19 during rest. The median RAS peaked at 148° during exercise, and was lowest at 135° during rest.

**Table 2:** The *p*-values from the Kruskal-Wallis tests of the comparisons of the ClassA metrics, across all five test epochs.

**ClassA metrics: *p*-values**	
**Metric**	***p*-value**
P^Q1^	**7.4 × 10^−5^**
P^Q2,4^	**1.2 × 10^−5^**
P^Q3^	**0.002**
RAS (°)	**1 × 10^−4^**

*Significant p-values are shown in bold (p ≤ 0.05)*.

**Figure 4:**
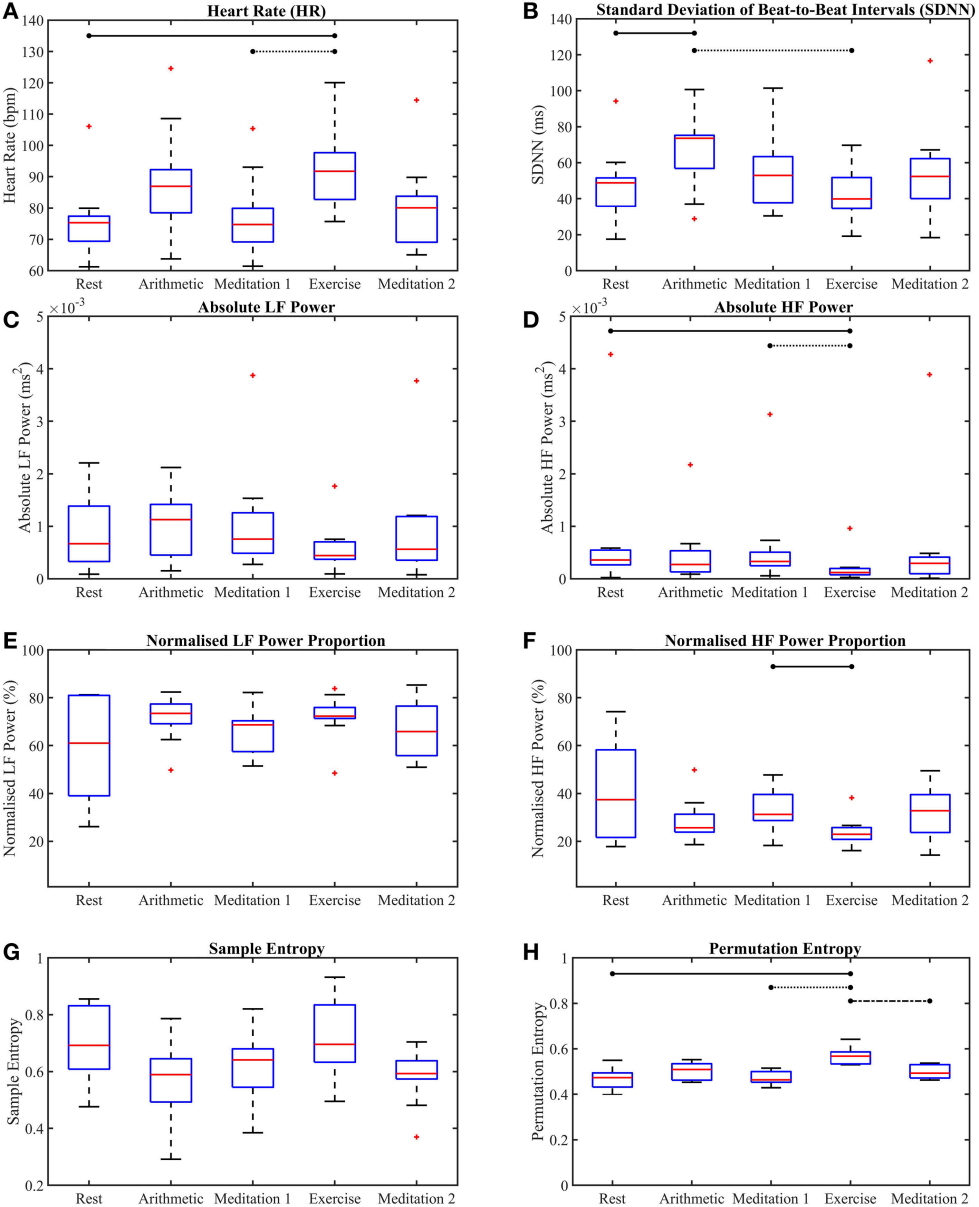
Box-plots of the traditional heart rate variability measures computed from all five test epochs. Heart rate **(A)**, the standard deviation of beat-to-beat intervals **(B)**, absolute power of the LF band **(C)**, absolute power of the HF band **(D)**, normalized power of the LF band **(E)**, normalized power of the HF band **(F)**, sample entropy **(G)**, permutation entropy **(H)**. The horizontal lines indicate statistically significant differences, and the red crosses indicate values which are 1.5 times outside the interquartile range of the boxplot.

**Figure 5:**
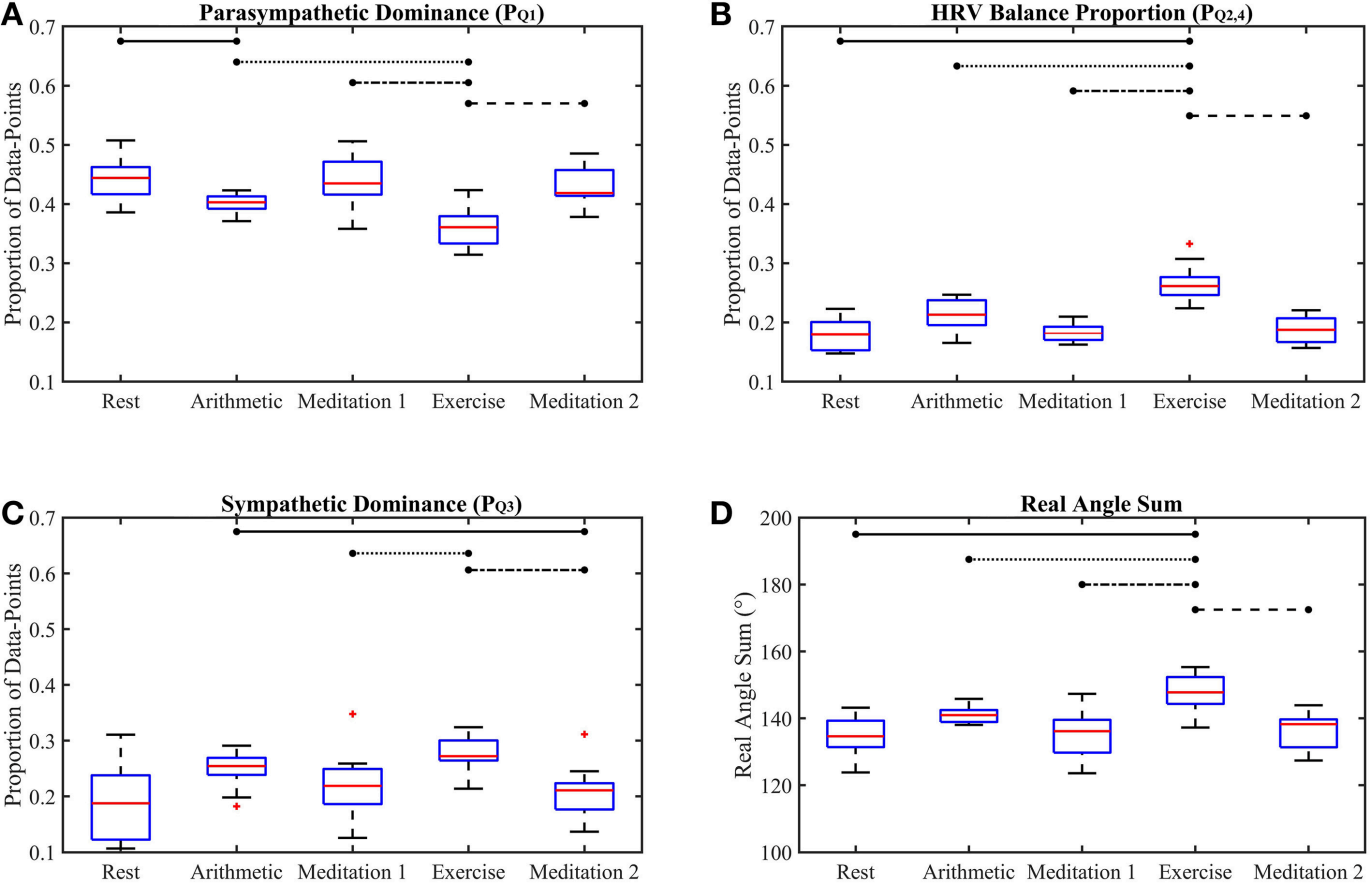
Box-plots of the proposed Classification Angle (ClassA) metrics computed from all five test epochs. The proportion of HRV rates of change representing cardiac deceleration **(A)**, HRV balance **(B)**, cardiac acceleration **(C)** and the Real Angle Sum **(D)**. The horizontal lines indicate statistically significant differences, and the red crosses indicate values which are 1.5 times outside the interquartile range of the boxplot.

Figures 4, 5 show the box-plots which display the traditional HRV measures and the ClassA metrics, respectively. Statistically significant differences between results from the different test epochs are indicated by horizontal lines, where the nodes on the lines specify the two epochs which were statistically different.

**Figure 6:**
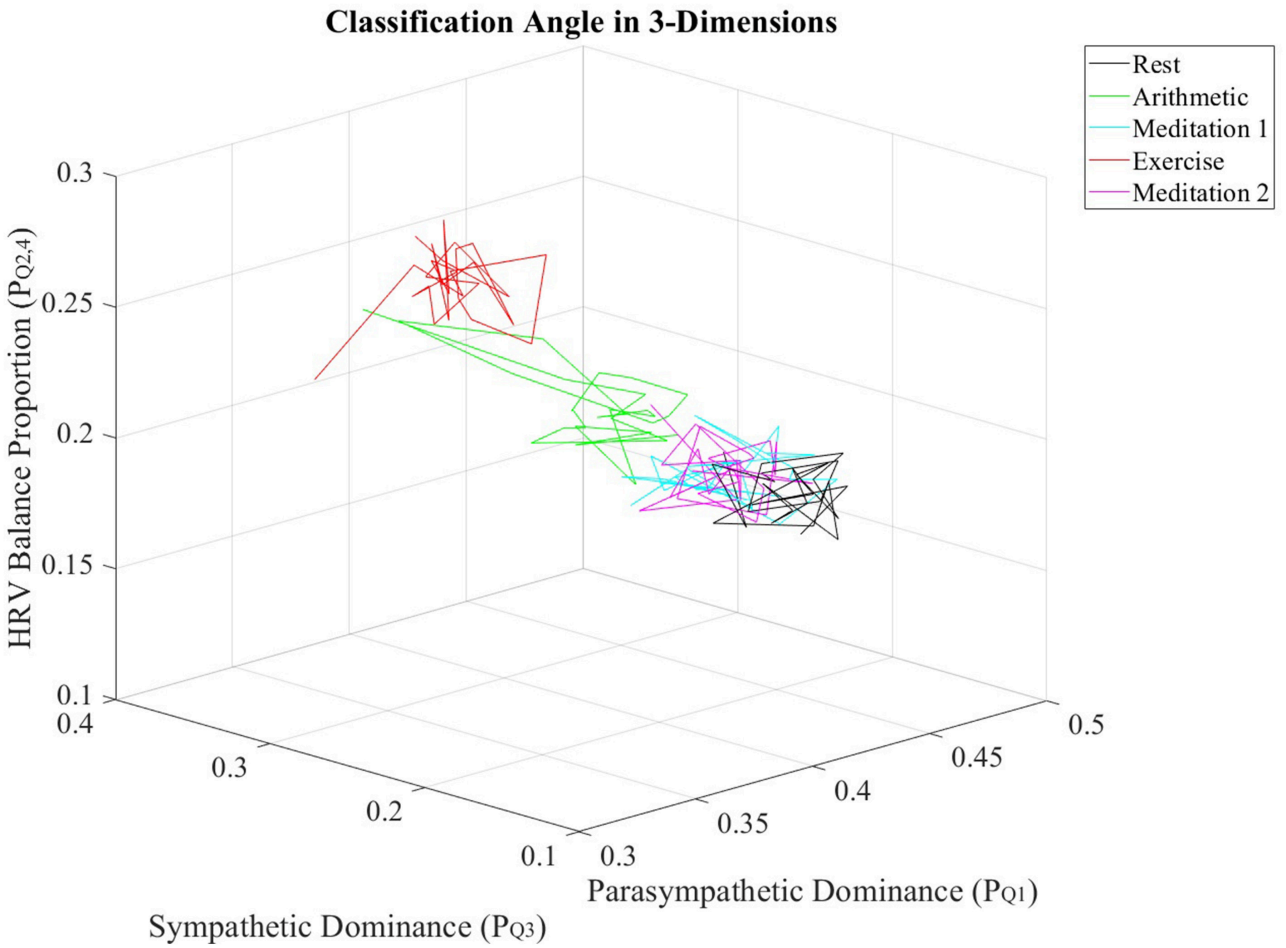
Trajectories of $P^{{}_{Q1}}$, $P^{{}_{Q2,4}}$ and $P^{{}_{Q3}}$ values, averaged across all subjects, for every epoch in the stress test.

Furthermore, the $P^{{}_{Q1}}$, $P^{{}_{Q2,4}}$, and $P^{{}_{Q3}}$ values from all subjects were individually averaged over nonoverlapping windows of 30 s in length, to produce averaged trajectories of $P^{{}_{Q1}}$, $P^{{}_{Q2,4}}$ and $P^{{}_{Q3}}$ for the stress test. Figure 6 displays the resulting trajectories.

### 4.2. Multiple Comparisons of Epochs of Stress and Epochs of No Stress

Tables 3, 4 display the *p*-values from the pairwise Kruskal-Wallis tests. The traditional HRV measures computed from the arithmetic epochs were compared to those from the baseline rest epochs, whilst the values from the exercise epochs were compared to those from the preceding meditation 1 epochs. All statistically significant *p*-values are shown in bold.

**Table 3:** Pairwise “stress vs. no stress” comparisons: *P*-values from the Kruskal-Wallis tests of traditional HRV measures.

**“Stress vs. No Stress” Kruskal-Wallis tests of traditional HRV measures: *p*-values**								
**Epochs**	**HR (*bpm*)**	**SDNN (*ms*)**	**aLF (*ms*^2^)**	**aHF (*ms*^2^)**	**nLF (%)**	**nHF (%)**	**SE**	**PE**
Rest vs. Arith.	0.028	0.041	0.36	0.55	0.20	0.23	0.049	0.096
Med. 1 vs. Exc.	**0.010**	0.17	0.13	**0.019**	0.13	**0.019**	0.15	**0.0002**

*Significant p-values are shown in bold (p ≤ 0.025, Bonferroni correction of 0.05/2)*.

**Table 4:** Pairwise “stress vs. no stress” comparisons: *P*-values from the Kruskal-Wallis tests of the ClassA metrics.

**“Stress vs. No Stress” Kruskal-Wallis tests of the proposed ClassA metrics: *p*-values**				
**Epochs**	**P^Q1^**	**P^Q2,4^**	**P^Q3^**	**RAS** (°)
Rest vs. Arith.	**0.0065**	**0.016**	**0.023**	**0.013**
Med. 1 vs. Exc.	**0.0012**	**0.0002**	**0.0082**	**0.0012**

*Significant p-values are shown in bold (p ≤ 0.025, Bonferroni correction of 0.05/2)*.

### 4.3. Correlations Between the Classification Angle Metrics and the Traditional Measures

Table 5 displays the Spearman rho correlations and the associated *p*-values from the correlation analyses of the ClassA metrics and the traditional measures. Significant correlations are shown in bold (*p* ≤ 0.0016, Bonferroni correction of 0.05/32).

**Table 5:** Spearman rho correlations Between the Classification Angle metrics and the traditional measures.

**“Stress vs. No Stress” Kruskal-Wallis tests of the proposed ClassA metrics: Spearman's Rho [*p*-value]**				
**Metric**	**P^Q1^**	**P^Q2.4^**	**P^Q3^**	**RAS (°)**
HR (*bpm*)	**−0.64[1.2 × 10^−6^]**	**0.62[2.8 × 10^−6^]**	**0.72[1.7 × 10^−8^]**	**0.60[7.5 × 10^−6^]**
SDNN (*ms*)	0.097[0.50]	−0.31[0.027]	−0.23[0.11]	−0.081[0.57]
aLF (*ms*^2^)	0.11[0.45]	−0.41[0.0031]	−0.16[0.27]	−0.083[0.57]
aHF (*ms*^2^)	0.40[0.0042]	**−0.56[3.19 × 10^−5^]**	**−0.48[4.3 × 10^−4^]**	−0.37[0.0093]
nLF (%)	**−0.60[6.5 × 10^−6^]**	0.29[0.04]	**0.78[0]**	**0.57[1.9 × 10^−5^]**
nHF (%)	**0.69[1.2 × 10^−7^]**	−0.42[0.0024]	**−0.85[0]**	**−0.66[3.7 × 10^−7^]**
SE	0.20[0.17]	0.16[0.26]	−0.38[0.0066]	−0.16[0.25]
PE	**−0.57[2.3 × 10^−5^]**	**0.92[0]**	**0.46[9.18 × 10^−4^]**	**0.55[4 × 10^−5^]**

*Significant p-values are shown in bold (p ≤ 0.0016, Bonferroni correction of 0.05/32)*.

## 5. Discussion

### 5.1. Performance of Traditional Measures

#### 5.1.1. Temporal Measures

The *p*-values displayed in Table 1 show that for separability across all five test epochs, the temporal measure of HR produced a statistically significant *p*-value of 0.013, and thus exhibited greater separability than SDNN.

Heart rate is the most basic cardiac measure to indicate the function of the autonomic nervous system (ANS), it is therefore not surprising that HR was the best performing of the temporal measures. The increased HR seen during the arithmetic test and exercise indicate a sympathetic stress reaction, which is to be expected amongst a study cohort of males—there is growing evidence to suggest that the feminizing hormone estrogen enhances parasympathetic activity, leading to females exhibiting parasympathetic reactions to stress (Dart et al., 2002; Adjei et al., 2018). It also comes as no surprise that HR outperformed SDNN when using an analysis window of 5 min, as the performance of SDNN has been found to be dependent upon data-length (Shaffer and Ginsberg, 2017). Shaffer and Ginsberg (2017) reviewed temporal, spectral and nonlinear cardiac metrics and concluded that SDNN produces meaningful results for data in which circadian rhythms are present. When using 24-h HRV, SDNN is known to be an excellent predictor of mortality and morbidity, whilst the accuracy of HR is unaffected by data-length (Shaffer and Ginsberg, 2017).

The box-plots shown in Figure 4 provide insight into the *p*-values displayed in Table 1. Figure 4A shows that, as expected, both mental and physical stress (the arithmetic test and exercise, respectively) caused increases in HR, with the median HR during exercise being nearly 5 *bpm* higher than that during the arithmetic test. However, the SDNN during the mental and physical stress epochs produced very different results, with the median SDNN during the arithmetic test being the highest of all the epochs at 74 *ms*, whilst the median SDNN during the exercise epoch was the smallest of all the epochs at 40 *ms*. The latter trend of stress-induced decreases in SDNN is the expected response to stress, as a low SDNN indicates a highly regular HRV (Kim et al., 2018). High regularity within HRV is synonymous with ill-health as it indicates a lack of quick adaptation of the ANS, which is required to face new challenges (Shaffer and Ginsberg, 2017). Therefore, the high SDNN seen during the arithmetic test indicates the activation of a stress response that retains the capacity to adapt to more stress. The disparity between the median SDNN from the arithmetic epoch and that from the exercise epoch also shows that the two forms of stress do not elicit the exact same response. Indeed, differences have been found in the physiological responses to physical and mental stress; for example, it has been found that mental stress causes a greater and faster release of the stress hormone cortisol, than exercise stress (Lovallo et al., 2006). The large SDNN seen during the arithmetic test indicates that HRV continued to vary during the test (Castrillión et al., 2017), and did not reach a plateau, whereas the low SDNN seen during exercise indicates that HRV reached a plateau, and ceased to vary.

Table 3 illustrates the good performance of HR in the separation of the different epochs of stress, as HR produced a statistically significant difference between the “stress vs. no stress” epochs (Bonferroni corrected *p*-value ≤ 0.025). The HR *p*-value for “Med. 1 vs. Exc.” was significant at 0.01, though the *p*-value for “Rest vs. Arith.” was only marginally significant, at 0.028; the *p*-values from the SDNN comparisons were not significant.

#### 5.1.2. Spectral Measures

The better separability of the five test epochs using the HF metrics compared to the LF metrics is remarkable. The poorest traditional measure was aLF, with a large and statistically insignificant *p*-value of 0.41, followed by nLF, with a smaller, but also statistically insignificant *p*-value of 0.37. Figures 4C,E verify this poor performance of the LF powers as they show that although the interquartile ranges of the measures decreased during exercise, the arithmetic and exercise boxes still overlapped with those from the other epochs. The HF boxes for exercise (shown in Figures 4D,F) had negligible overlap with those of the other epochs, which implies that the HF band provides greater information pertaining to physical stress than the LF band. The difference in the effectiveness of the LF and HF bands to discern physical stress also suggests that LF and HF do not have a strictly reciprocal relationship.

Whilst the *p*-values from the pairwise LF comparisons were not significant, the *p*-values from the pairwise HF comparisons of “Med. 1 vs. Exc.” were significant, at 0.019 (although those from the HF comparisons of “Rest vs. Arith.” were not statistically significant). As PNS activity is known to diminish at the onset of physical stress, but not necessarily during mental stress, the small *p*-values for HF indicate a large difference in the cardiac dynamics of “Med. 1 vs. Exc.,” and hence, could support the theory that HF is heavily influenced by the PNS (Malik et al., 1996).

The large overlaps amongst the LF boxes in Figures 4C,E indicate relatively small changes in LF throughout the stress test. The lack of separability of the epochs provides insight into whether LF represents the activity of the SNS, since a measure that truly represented the SNS would undoubtedly separate the epochs of stress from the epochs of rest and meditation. The poor performance of LF proves that it cannot be solely controlled by the SNS, and must be influenced by other physiological functions, supporting the theories which suggest that LF is influenced by multiple factors, ranging from the PNS and slow breathing, to vascular contractions (Brown et al., 1993; Kenwright et al., 2008; Billman, 2013).

The suggestion that the LF band is influenced by many physiological functions is further supported by the finding that there was a considerably greater proportion of power in the LF band, compared to the HF band. The greater power in the LF band is shown in Figures 4C,E, and when compared to Figures 4D,F, the greater power of LF is noticeable as HF would be expected to dominate during the epochs of rest and meditation.

In summary, aLF, aHF, nLF and nHF did not perform as well as the simpler methods of HR and SDNN in the discernment of stress across all five test epochs, although HF was able to discern physical stress from no stress in the pairwise epoch comparisons. However, the LF metrics of aLF and nLF were not able to significantly discern either of the stress epochs from the preceding epochs of no stress; the prolific use of LF in the literature to assess ANS dynamics therefore appears erroneous.

#### 5.1.3. Nonlinear Measures

The performances of SE and PE in the discernment of stress were very different to one another. Despite the widespread use of SE, its performance was not as good as that of the lesser known PE algorithm. Sample entropy was able to significantly between the test epochs across all five epochs, but did not produce any significant results in the pairwise epoch comparisons. Moreover, the trend in Figure 4G suggests that mental and physical stress elicited different SE responses; mental stress, in the form of the arithmetic test, produced comparatively low SE values, whilst physical stress, in the form of exercise, produced comparatively high SE values. The reductions in SE during mental stress are supported by findings from Vuksanovic and Gal (2007), Williamon et al. (2013), and Chanwimalueang et al. (2016). In contrast, PE, produced higher values during both the arithmetic and exercise epochs. The differing trends in the SE and PE results suggest that SE and PE were not measuring the same dynamic within the signals. Although sample entropy was introduced as a measure which reaches a minimum when a signal is regular (Pincus, 1991; Richman and Moorman, 2000), and PE was introduced as a measure which is at a minimum when a signal is predictable (Bandt and Pompe, 2002), the results presented here suggest that they are measuring different signal dynamics. For example, the multiscale sample entropies from 1/f noise (so-called pink noise) have been found to remain stable as temporal scale increases (Costa et al., 2002), whilst the multiscale permutation entropies from pink noise have been found to decrease as temporal scale increases (Deng et al., 2017). Whilst these differences have been cited as limitations of the PE algorithm in measuring entropy (Deng et al., 2017), the greater separability of epochs offered by PE in this present study, and the results produced by the implementation of a symbolic method very similar to PE in Porta et al. (2007) suggest that the identification of patterns in HRV signals is useful in assessing stress.

Across all epochs, PE produced the most significant *p*-value of the traditional measures at *p* = 9.3 × 10^−5^, and along with HR and the HF metrics, was able to produce a statistically significant *p*-value in the comparison of “Med. 1 vs. Exc.,” though the *p*-value from the comparison of “Rest vs. Arith.” was not significant. It must also be noted that in spite of the good performance of PE in discerning stress, neither PE nor SE were able to provide information on the functioning of the SNS and PNS.

It is noticeable that none of the traditional measures were able to significantly discern mental stress from no stress.

### 5.2. Performance of the Proposed Classification Angle Metrics

The *p*-values displayed in Table 2 demonstrate the effectiveness of the ClassA metrics in distinguishing between the epochs of the stress test, with exceptionally small *p*-values of 7.4 × 10^−5^, 1.2 × 10^−5^ and 1 × 10^−4^ for $P^{{}_{Q1}}$, $P^{{}_{Q2,4}}$ and RAS, respectively, and a larger, but still significant *p*-value of 0.002 for $P^{{}_{Q3}}$. The significance of these *p*-values is verified by Figure 5, which shows that the boxes from the stress epochs had negligible overlap with those from the epochs of no stress. It is also evident that the boxes related to exercise also had negligible overlap with the boxes related to the arithmetic test, with the exercise epoch results being the most distinct from the rest and meditation epochs.

Figure 5A shows that both mental and physical stress were associated with a reduction in $P^{{}_{Q1}}$; physical stress caused a greater reduction. Taking $P^{{}_{Q1}}$ to represent cardiac deceleration, and hence parasympathetic dominance, the stress-induced decreases of $P^{{}_{Q1}}$ show that PNS withdrawal contributed greatly to the stress-induced increase in HR. The conventional understanding of the stress response often assumes a reciprocal relationship between the PNS and SNS, where rises in HR are caused by coupled SNS activation and PNS withdrawal (Billman, 2013). However, assuming that $P^{{}_{Q3}}$ represents cardiac acceleration, and hence SNS dominance, the box-plots of $P^{{}_{Q3}}$ in Figure 5C show increases in cardiac acceleration during the stress epochs which were smaller than the corresponding decreases in $P^{{}_{Q1}}$. For example, $P^{{}_{Q1}}$ was 0.4 and 0.36, respectively, during the arithmetic and exercise epochs, whilst the corresponding $P^{{}_{Q3}}$ values were 0.25 and 0.27, respectively. An increase in HR caused by coupled PNS withdrawal and SNS activation, is termed *reciprocal sympathetic activation* (Berntson et al., 1991). However, greater parasympathetic withdrawal is not often associated with the stress response, though an uncoupled parasympathetic withdrawal response to physical stress had been widely reported as far back as 1966, when Robinson et al. (1966) reported initial heart rate increases caused by parasympathetic withdrawal in response to exercise in the supine position. Robinson et al. (1966) only reported sympathetic activation after prolonged exercise. Though the speed of parasympathetic nerves (≤ 1 s) is only marginally faster than that of sympathetic nerves (≥5 s) (Nunan et al., 2010), the aggregated effects of a slower release of sympathetic hormones, such as norepinephrine, could explain the predominance of PNS withdrawal in the initial HR response to exercise (Gordan et al., 2015). The $P^{{}_{Q1}}$ and $P^{{}_{Q3}}$ results reported here could therefore reflect this predominance of PNS withdrawal over SNS activation during a short bout of exercise. *The ClassA signal metrics can thus be used to identify whether the activities of the PNS and SNS are coupled*.

The phenomenon of HR increases due to greater PNS withdrawal is supported by the biological understanding of cardiac dynamics. Biologically, intrinsic HR is in the region of 100 *bpm*, but neural or hormonal influences slow the contractions of the heart from its intrinsic rate to a typical resting HR of 60-90 *bpm* (Gordan et al., 2015). Therefore, sympathetic activation is not required to initially increase HR from resting levels, since PNS withdrawal alone is sufficient to increase HR by as much as 40 *bpm* (Gordan et al., 2015). The activation of the SNS to increase HR in the “fight-or-flight” response may not be as instant as it is often assumed to be.

The box-plots shown in Figure 5B display the opposite trend to that seen in Figure 5A, and a similar trend to that seen in Figure 5C. This similarity between $P^{{}_{Q2,4}}$ and $P^{{}_{Q3}}$ is unexpected as $P^{{}_{Q2,4}}$ was computed from the first temporal scale of HRV, whilst $P^{{}_{Q3}}$ was computed from the seventh temporal scale. Nevertheless, this similarity suggests that whilst the sympathetic nerves are slow acting, their effect can be seen beat-to-beat, quantified by $P^{{}_{Q2,4}}$.

The separation abilities of $P^{{}_{Q1}}$, $P^{{}_{Q2,4}}$, $P^{{}_{Q3}}$ and RAS were verified through the *p*-values obtained from the pairwise comparisons between each epoch of stress and its preceding epoch of no stress (see Table 4). It is evident that $P^{{}_{Q1}}$, $P^{{}_{Q2,4}}$, $P^{{}_{Q3}}$ and RAS were able to separate the states with statistical significance (Bonferroni corrected *p*-value ≤ 0.025). Noticeably, $P^{{}_{Q3}}$ produced the highest *p*-values of all the measures shown in Table 4, although they were still statistically significant. This comparatively poorer performance of $P^{{}_{Q3}}$ could be due to the choice to compute $P^{{}_{Q3}}$ from the seventh temporal scale of HRV. Heuristically, it was found that temporal scale seven provided the best compromise between the ability to capture longer-range SNS dynamics, whilst still retaining high temporal resolution. This trade-off in deciding upon the temporal scale at which to compute $P^{{}_{Q3}}$ is a limitation of the ClassA framework.

The separation ability of the fourth ClassA metric of RAS was next verified. The RAS metric provides a single value to describe the overall trend of HRV and although, if used alone, it could not indicate the degree of PNS and SNS dominance, it was able to separate the two stress epochs from the preceding epochs of no stress with significant *p*-values of 1.3 × 10^−2^ and 1.2 × 10^−3^ (Bonferroni corrected *p*-value ≤ 0.025). The RAS results shown in Figure 5D indicate that during rest and meditation, HRV was largely balanced but tended toward increasing (cardiac deceleration), whilst during stress, HRV was still largely balanced, but tended toward decreasing (cardiac acceleration). The ability of RAS to quantify the trend of the dynamics within a signal with a single value is unique. This metric therefore has the potential to be employed when stress must be identified, irrespective of the underlying autonomic mechanisms; this is not dissimilar to the use of the entropy measures to detect stress. Nevertheless, when compared to the entropy measures, RAS has the advantage that it is computationally inexpensive.

The computational efficiency of the ClassA framework is extremely advantageous as it enables an increase in the temporal resolution of stress studies. Not only have the results presented here demonstrated the effectiveness of ClassA on 10 s of HRV data to assess PNS dynamics, and when evaluated on 60-s, to assess SNS dynamics, but also, the framework is not mathematically complicated, and could be evaluated and fine-tuned by a clinician or practitioner who is new to programming.

The intuitive computation of the ClassA metrics is reflected in the results from the correlation analyses displayed in Table 5, where it is shown that all four of the ClassA metrics were significantly correlated to heart rate. The metric $P^{{}_{Q1}}$ produced a significant negative correlation with heart rate, whilst the other three ClassA metrics produced significant positive correlations. However, as already outlined, the ClassA metrics offered greater overall separability of the test epochs compared to heart rate. All four ClassA metrics were also significantly correlated to the results from the less computationally efficient PE analysis. Furthermore, unlike the one-dimensional analyses offered by both heart rate and PE, the ClassA metrics provided a multidimensional analysis of autonomic function.

The multidimensional separation ability of the ClassA metrics are demonstrated in Figure 6, which show that the three metrics of $P^{{}_{Q1}}$, $P^{{}_{Q2,4}}$, and $P^{{}_{Q3}}$ were able separate the epochs of stress from those of rest and meditation. The three-dimensional plot of the averaged trajectories of these metrics, across all subjects throughout the stress test, demonstrates that there are clear regions within the plot which represent the different stress states. This three-dimensional representation therefore has the potential to be used to identify an individual's psychophysiological status.

A limitation of this current study is that the respiratory rates of the subjects were not recorded, so the effect of respiration on the HRV signals could not be evaluated. In future studies, respiration must be recorded to enable an assessment of all the stress measures in the absence of respiratory effects. Also, the effectiveness of the ClassA framework to discern stress should be validated on female subjects. Females have been found to exhibit a different stress response to that seen in males, whereby, as mentioned in section 5.1.1, high levels of estrogen have been linked to enhancing PNS activity (Dart et al., 2002). Furthermore, since estrogen levels in females are dependent on the menstrual cycle, a stress study including females will have to involve analyses of the subjects based on their age and stage of the menstrual cycle at the time of participation. Such a detailed and thorough study will enable the assessment of the representation of stress in females using the ClassA framework, and is beyond the scope of this work.

## 6. Conclusion

The Classification Angle (ClassA) framework has been introduced to accurately capture stress-specific dynamics of the autonomic nervous system using heart rate variability (HRV). This has been achieved based on a second-order-difference-plot of HRV, which makes it possible to assess cardiac dynamics. The framework has been shown to identify which branch of the autonomic nervous system is dominating, without inheriting any of the controversies associated with the low- and high- frequency components of HRV. The Classification Angle framework is comprehensive and has been designed to produce four metrics to assess autonomic dynamics.

All four ClassA metrics have been able to distinguish between epochs of mental stress and physical stress, and the preceding epochs of no stress; this was established over experiments on 10 males, with statistically significant *p*-values in the range of 2 × 10^−4^ to 2.3 × 10^−2^ (Bonferroni corrected *p*-value ≤ 0.025). Out of the standard temporal, spectral and nonlinear HRV metrics (heart rate, standard deviation of beat-to-beat intervals, low frequency and high frequency components, sample entropy and permutation entropy of HRV) applied on the same data, heart rate, the high frequency components of HRV and permutation entropy were able to discern between epochs of physical stress from the preceding epochs of no stress, with *p*-values in the range of 2 × 10^−4^ to 1 × 10^−2^. None of the traditional measures have been able to discern between the epochs of mental stress and the preceding epochs of no stress.

Furthermore, unlike the traditional heart rate variability measures, the proposed classification angle metrics offer a three-dimensional interpretation of parasympathetic and sympathetic dynamics, together with the nature of their interaction, e.g., uncoupled or coupled. *An HRV measure that can indicate whether the activities of the sympathetic and parasympathetic nervous system are coupled has the potential to transform quantitative stress research, with the ClassA framework having been verified as one such metric*.

In summary, the proposed ClassA framework has been demonstrated to be robust and accurate in discerning stress from periods of no stress, and offers a three-dimensional analysis; its unique and desirable features include operations on nonstationary data, and its metrics admit physiological interpretation. The framework is also computationally inexpensive, and can be computed over as little as 10 s of heart rate variability data, a substantial increase of temporal resolution over the current 5-min standard.

## Ethics Statement

Approval for physiological sensing for stress assessment has been granted by the Imperial College Healthcare Trust Clinical Governance Department. All subjects gave full consent in accordance with the Declaration of Helsinki.

## Author Contributions

The physiological data were recorded by TA and WvR. The data analysis was completed by TA, TN, and TC, under the supervision of DM. The paper was written by TA and DM.

### Conflict of Interest Statement

The authors declare that the research was conducted in the absence of any commercial or financial relationships that could be construed as a potential conflict of interest.

## 7. Appendix

**Algorithm 1:** Sample Entropy

A windowed signal, *x*, of length *N* is segmented using an embedding dimension, *m*, to create (*N*−*m*+1) segments. Each such segment, *X*_*m*_, is of length *m*. In this study, *m* was defined as *m* = 2. A tolerance level of *r* = 0.15 × *std*, where *std* is the standard deviation in the data window, is defined. The maximum difference, *d*_*max*_, between the scalar components of two consecutive segments, *X*_*m*_(*i*) and *X*_*m*_(*j*), is computed as (8) $$\begin{array}{r} d_{max}=\underset{k=0,\ldots ,m-1}{max}\left(\left|x\left(i+k\right)-x\left(j+k\right)\right|\right) \end{array}$$ For each *X*_*m*_(*i*), the event *d*_*max*_<*r* is defined as a match, and a count of such matches is denoted by *A*_*i*_. The probability of matches, $A_{i}^{m}\left(r\right)$, for *X*_*m*_(*i*) is calculated as (9) $$\begin{array}{r} A_{i}^{m}=\frac{A_{i}}{N-m-1} \end{array}$$ Note: The denominator, *N*−*m*−1, ensures the segment *X*_*m*+1_(*i*) can be included in the computation of the probability. Then, the sum of the probability of matches for all segments, Φ, is defined as (10) $$\begin{array}{c} \Phi^{m}(r)=\frac{\sum_{i=1}^{N-m}A_{i}^{m}(r)}{N-m} \end{array}$$ The embedding dimension, *m*, is increased to (*m*+1), and Step 1 to Step 5 are repeated; the sum of the probability of matches for all segments when *m* = *m*+1 are defined as Ψ, and the sample entropy, *SE*(*m, r*), is computed as (11) $$\begin{array}{r} SE\left(m,r\right)=ln\left(\frac{\Phi }{\text{Ψ}}\right) \end{array}$$ Sample entropy for *x* is computed such that one SE value is obtained for each data window.

**Algorithm 2:** Permutation Entropy

A windowed signal, *x*(*n*), of length *N* is segmented using an embedding dimension, *m*, to create (*N*−*m*+1) segments. Each such segment, *X*_*m*_, is of length *m*. In this study, *m* was defined as *m* = 6. The elements in *X*_*m*_ are ranked into ascending order, *x*(*n*+*j*_1_−1) ≤ *x*(*n*+*j*_2_−1) ≤ ⋯ ≤ *x*(*n*+*j*_*m*_−1) where *j* is the time index of each element. Note that if *x*(*n*+*j*_1_−1) = *x*(*n*+*j*_2_−1), the segments are ordered in accordance with their time indices. The time indices of the re-ordered vectors are then used to map each segment onto a symbol, (12) $$\begin{array}{r} A\left(n\right)=\left(j_{1},j_{2},\ldots ,j_{n}\right) \end{array}$$ The total number of unique symbols is denoted as *k*, and the relative frequency of each unique symbol, is found as *P*_*n*_. Permutation entropy (PermEn) is computed as the Shannon entropy of the relative frequencies of the unique symbols, (13) $$\begin{array}{r} PermEn\left(m\right)=\overset{k}{\underset{n=1}{\sum }}P_{n}lnP_{n} \end{array}$$ The computed *PermEn* is then normalized to enable comparisons with results from signals of different lengths. The normalized *PermEn* is denoted by PE, and is given by, (14) $$\begin{array}{r} PE=\frac{PermEn\left(m\right)}{ln\left(m!\right)} \end{array}$$
